# Characterization of laser-driven proton acceleration from water microdroplets

**DOI:** 10.1038/s41598-019-53587-3

**Published:** 2019-11-20

**Authors:** Georg A. Becker, Matthew B. Schwab, Robert Lötzsch, Stefan Tietze, Diethard Klöpfel, Martin Rehwald, Hans-Peter Schlenvoigt, Alexander Sävert, Ulrich Schramm, Matt Zepf, Malte C. Kaluza

**Affiliations:** 1 Institut für Optik und Quantenelektronik, Friedrich-Schiller-Universität Jena, Max-Wien-Platz 1, D-07743 Jena, Germany; 2 Helmholtz-Institut Jena, Fröbelstieg 3, D-07743 Jena, Germany; 3 Helmholtz-Zentrum Dresden-Rossendorf (HZDR), Bautzner Landstraße 400, D-01328 Dresden, Germany; 4 Technische Universität Dresden, D-01062 Dresden, Germany

**Keywords:** Laser-produced plasmas, Plasma-based accelerators

## Abstract

We report on a proton acceleration experiment in which high-intensity laser pulses with a wavelength of 0.4 μm and with varying temporal intensity contrast have been used to irradiate water droplets of 20 μm diameter. Such droplets are a reliable and easy-to-implement type of target for proton acceleration experiments with the potential to be used at very high repetition rates. We have investigated the influence of the laser’s angle of incidence by moving the droplet along the laser polarization axis. This position, which is coupled with the angle of incidence, has a crucial impact on the maximum proton energy. Central irradiation leads to an inefficient coupling of the laser energy into hot electrons, resulting in a low maximum proton energy. The introduction of a controlled pre-pulse produces an enhancement of hot electron generation in this geometry and therefore higher proton energies. However, two-dimensional particle-in-cell simulations support our experimental results confirming, that even slightly higher proton energies are achieved under grazing laser incidence when no additional pre-plasma is present. Illuminating a droplet under grazing incidence generates a stream of hot electrons that flows along the droplet’s surface due to self-generated electric and magnetic fields and ultimately generates a strong electric field responsible for proton acceleration. The interaction conditions were monitored with the help of an ultra-short optical probe laser, with which the plasma expansion could be observed.

## Introduction

Since the first detection of laser-accelerated protons with kinetic energies of several tens of MeV around the turn of the millenium^1–4^, many experiments in the field of laser-driven proton acceleration have been performed by focussing laser pulses (typical central wavelength between 0.8 μm and 1 μm) to an intensity *I* > 10^18^ W/cm^2^ onto a solid-state target, e.g. a metal foil with a thickness of some μm. The laser ionizes the foil’s front and accelerates electrons through the foil. On the foil’s rear surface they form an electron sheath leading to a charge separation field in the order of MV/μm. This field accelerates ions and protons from the foil’s back along its normal direction, hence giving this acceleration process the name Target Normal Sheath Acceleration (TNSA)^5^. The acceleration process takes place on a micrometer scale, which allows for the realization of a compact accelerator as part of a high-intensity laser facility for various applications. It has already been demonstrated that it is possible to use laser-accelerated protons for stress testing of materials^6^, investigation of cultural heritage^7^, as a front-end for conventional accelerators^8,9^ or to probe transient electromagnetic fields with ps temporal and μm spatial resolution^10^. Potential future applications include biomedical ones such as hadron therapy^11,12^. For all these applications, a reliable and controllable source of energetic protons is crucial. In addition, the availability of proton pulses with a high repetition rate, at least of the order of 10 Hz but also higher, would be desirable.

While the application of the typical foil targets for proton-acceleration experiments aiming at high repetition rates is possible, it is rather challenging, since the foil destroyed by the laser needs to be replaced by a fresh target after each shot^13^. Such new targets need to be aligned with very high accuracy to the laser focus in order to allow for reproducible and controllable generation of proton pulses. In addition to the challenges of the exact target alignment, the foil’s typical lateral extent of several mm and more allows electrons to escape from the interaction region parallel to the foil surface^14^, which may decrease the efficiency of the proton acceleration process.

An approach to avoid this loss in efficiency is the use of mass-limited targets that have no physical connection to their environment. Such targets that can be used on a single shot basis are e.g. plastic spheres levitated in a Paul Trap^15–18^. When it comes to experimental statistics or applications, however, cost-effective spherical targets available with a high repetition rate are more useful. Water microdroplets generated by a commercial nozzle with a repetition rate of about 1 MHz have been used in experiments performed at the Max Born Institut in Berlin, but only ions with kinetic energies of less than 1 MeV per nucleon could be accelerated^19–23^, which was attributed to the temporal intensity contrast (TIC) of their laser system. Additionally the actual interaction conditions between laser and droplet could so far not be observed. Therefore, it is so far not known which degrees of accuracy are required for the alignment of the targets, i.e. the single spheres with respect to the laser pulse or which TIC is necessary to optimize laser-driven proton acceleration. In parallel with the need for higher kinetic energies, the goal is to better understand the laser-plasma interaction itself. This includes, for example, the angle of incidence and the position of the laser on the target, the existence and extent of a pre-plasma and the evolution of the plasma expansion. All this can be investigated using a synchronized optical probe beam^24–29^. These investigations are pivotal for optimizing the ion acceleration process and evaluating the potential of such high repetition rate, mass-limited targets for future applications.

In this paper, we show the influence of the droplet position relative to the laser focus on the maximum kinetic energy of protons accelerated in laser forward direction by laser pulses with ultra-high TIC. This ultra-high TIC was achieved by frequency-doubling the laser pulses to a wavelength of 0.4 μm. In addition we investigated the influence of a pre-pulse on the maximum proton energy. The pre-pulse's arrival time relative to the main laser pulse could be controlled. The interaction conditions were observed with high spatial and temporal resolution using a few-cycle optical probe system^27,30^.

With the help of two-dimensional particle-in-cell (2D-PIC) simulations we show that a pre-plasma, introduced by the pre-pulse, enhances the laser absorption of the plasma, which results in an increase of the maximum proton energy for normal laser incidence. However, proton energies comparable on average, but higher for individual shots, were achieved under grazing laser incidence without a pre-plasma present. For these conditions, we found that an electron current is produced close to the droplet’s surface generating electric and magnetic fields that lead to a confinement of the surface current^31–35^ and thus an increase in the electric field that primarily drives the proton acceleration.

## Experimental Setup

**Figure 1:**
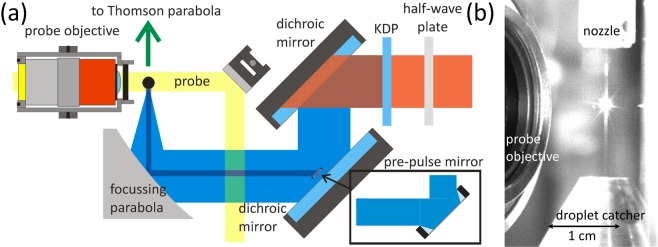
(**a**) Schematic showing the experimental setup (not to scale). (**b**) Picture of the water jet illuminated by the attenuated laser. The water is collected by the heated droplet catcher which is evacuated with a seperate vacuum pump.

The experiment has been carried out with the Jena Ti:sapphire (JETI) 40 TW laser system at the Institut für Optik und Quantenelektronik in Jena, Germany. The laser pulses entering the target chamber had a spectrum centered around 800 nm, a pulse energy of ~750 mJ and a full width at half maximum (FWHM) pulse duration of ~35 fs. To be able to significantly suppress pre-plasma fomation the laser pulses were frequency-doubled inside the target chamber by a KDP crystal increasing the TIC^36^. The pulse duration of the frequency-doubled laser pulses was not measured in the experiment, but simulated to have a duration of ~42 fs^37^. The horizontally polarized pulses were focused by an aluminum off-axis parabola with a focal length of 101.6 mm (~F/1.8) into a focal spot with a size of *A* ≈ 1.1 µm^2^. Within this area, the intensity remained higher than 50 % of the peak intensity. This focal spot contained *q* ≈ 20 % of the total 2*ω* laser pulse energy of ~90 mJ. Since the laser’s focus could not be measured at full intensity, *A* and *q* were obtained by imaging the focus with low laser energy and averaging over several of these low intensity focal spots. This gives an approximate intensity of *I* ≈ 4 · 10^19^ W/cm^2^ on the target, which corresponds to ${a}_{0}=\sqrt{(I\cdot {\lambda }^{2})/(1.37\cdot {10}^{18}\,{\rm{W}}/{{\rm{c}}{\rm{m}}}^{2}{\cdot \mu {\rm{m}}}^{2})}\approx 2.1$ with *λ* = 0.4 μm. To suppress the remaining radiation at the fundamental wavelength, two dichroic mirrors were installed in front of the focussing parabola (Fig. 1). The resulting TIC is shown in the Supplemental Material. In order to produce a controlled pre-plasma with an artificial pre-pulse, a movable half-inch mirror was installed in front of the second beam mirror. Due to its smaller diameter, this pre-pulse produced a much larger focal spot than the main pulse^37,38^. The approximate pre-pulse intensity was 10^16^ W/cm^2^. When necessary, the pre-pulse could be isolated from the main pulse with a circular aperture of ~8 mm diameter.

As targets, water droplets with a diameter of ~20 μm were used, which were generated with a nozzle by Micro Jet Components, Sweden. These nozzles are equipped with a piezo element driven by a high-frequency voltage that has been synchronized with the laser repetition frequency, resulting in a controlled breakup of the water jet emitted from the nozzle into droplets relative to the focus position and timing of the main laser pulse. The falling droplets were collected by a seperately pumped, heatable droplet catcher with an entrance aperture diameter of ~0.5 mm. To control the droplet position and to observe the interaction between laser and droplets, the broadband few-cycle probe pulse of the JETI system was used^27,30^. The spectrum of this probe pulse spanned from 525 nm to 925 nm. Between the objective and the camera a bandpass filter with (710 ± 20) nm transmission was positioned to minimize the signal from the broadband plasma emission.

The kinetic energy of the accelerated protons was measured in the pump laser’s forward direction with a Thomson parabola spectrometer equipped with a multichannel plate (MCP) detector. Its 1 mm diameter aperture was positioned 97 cm from the droplets so that the spectrometer covered a solid angle of ≈ 0.8 µsr. The magnetic field region in the parabola with an effective field strength of ~570 mT was about 10 cm long. The electric field region generated by a plate capacitor with a length of 20 cm which was placed between the magnets in the iron yoke was set to ~4.5 kV/cm. The distance between MCP and the end of the field region was 5 cm while the distance between MCP and aperture was 35.5 cm. For a proton energy of 2 MeV the size of the projected aperture in the detector plane resulted in an energy resolution of about Δ*E* ≈ 0.2 MeV.

## Experimental Results

**Figure 2:**
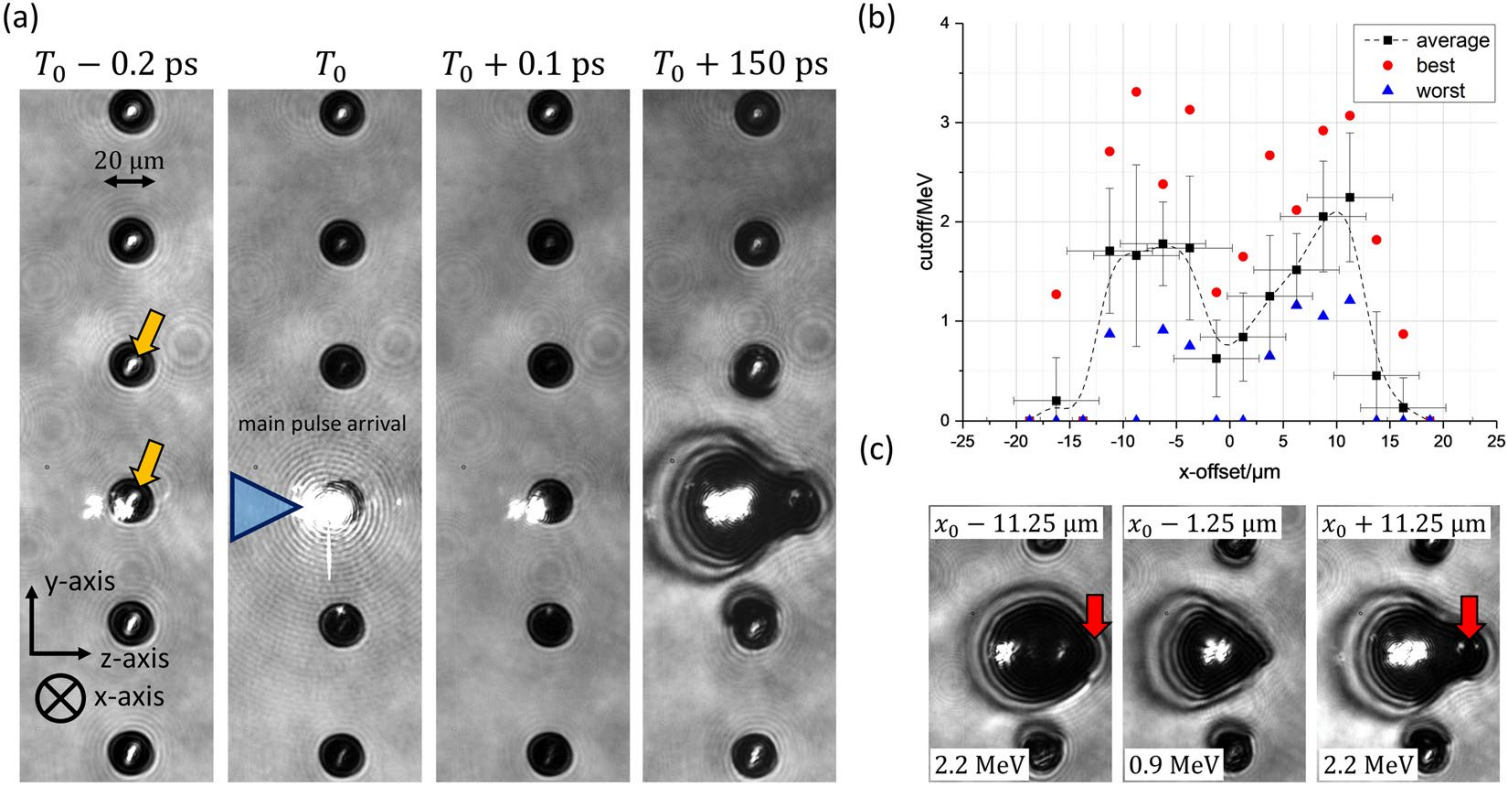
Exemplary images of the laser-droplet interaction for four different delays between probe and main pulse arrival *T*_0_ are shown in (**a**). The orange arrows point at the probe light which is focused by the transparent droplets before the laser arrives at *T*_0_ − 0.2 ps. The main laser illuminates the central droplets from the left. In (**b**) the maximum proton energy is shown as a function of the position of the droplets relative to the laser focus position along the polarization axis. The black squares are averaged values with standard deviation of 10 consecutive shots taken at each position, except for *x* = +16.25 μm (11 shots), *x* = +18.75 μm (9 shots) and *x* = −16.25 μm (15 shots). The whole scan consists of 165 consecutive shots between *x* = −18.75 μm and *x* = +18.75 μm. Every single shot is used in the calculation of the mean values and the corresponding standard deviations. Shots with no signal at or above the Thomson parabola’s low energy threshold of 0.4 MeV were included as 0 MeV in the calculation of the mean values and standard deviations. Red dots and blue triangles represent the highest and lowest proton energies of the different positions, respectively. If the blue triangle at one position represents a value higher than 0 MeV, all of the shots taken at this certain position, for example at *x* = 11.25 μm, produced a signal on the Thomson parabola’s MCP. Since the droplets’ absolute positions relative to the laser pulse in the *x*-axis could not be measured directly, it was estimated from the symmetry of the scan. Details about the error bars along the x-axis can be found in the Supplemental Material. (**c**) Shows exemplary images of the illuminated droplet’s expansion for different positions 150 ps after the arrival of the main laser pulse.

With the optical probe we investigated the interaction of the laser pulse with the droplets for different temporal delays between probe and main pulse (Fig. 2(a)). For the first shown time step, *T*_0_ − 0.2 ps, i.e. 0.2 ps before the arrival of the main pulse at *T*_0_, all droplets are transparent for the probe and act as lenses for the probe’s light, generating bright spots in the center of the droplets’ images. The bright light on the left of the central droplet visible in every image shown is due to plasma radiation emitted after the main pulse’s arrival. It is visible on the image since the camera’s exposure time was much longer (~μs) than the probe pulse’s duration. As soon as the main pulse arrives it ionizes not only the central droplet but also its neighbors as evidenced by the fact that they become opaque to the probe. 0.1 ps later, which corresponds to the time scale on which the ion acceleration should take place, the central droplet has still kept its shape and size. 150 ps later, however, a significant expansion is visible. The expansion of the central droplet most likely influences several neighboring droplets on a nanosecond timescale, inhibiting the use of every single droplet as an ion source. Therefore, the maximum repetition rate in an experiment is likely below ~1 MHz, but most likely still in the 10 kHz to 100 kHz range. Although this expansion process is no longer directly related to the MeV-ion acceleration process it does provide an insight into the conditions under which the droplets were irradiated by the laser pulses and can be correlated to the maximum proton energy. This will be described below.

In order to investigate how the pump laser interacts with the droplets we varied their position along the laser’s polarization axis (*x*-axis) while measuring the maximum proton energy in laser forward direction and taking side-view images of the droplet expansion 150 ps after the main pulse’s arrival. In Fig. 2(b) the maximum proton energy is plotted against the droplet position along the *x*-axis. Since we could not measure the exact droplet position relative to the central laser axis, the zero offset was set relative to the plot’s axis of symmetry. It can be seen that the proton energy is lowest when the droplets were hit centrally and increases by more than a factor of 2 when the droplets’ sides were illuminated. A comparable behavior can be observed for the droplet expansion in Fig. 2(c). When the droplets were irradiated on their sides (left and right image) a strong plasma expansion took place and a bulge is visible on the right side of the droplets (red arrows). In contrast to that, the plasma expanded less in laser forward direction when the droplets were illuminated centrally (central image). In the Supplemental Material (Fig. 8) all side-view images captured at *x* = +1.25 μm are shown. There, it can be seen that the size of the plasma expansion at this late time directly correlates with the maximum proton energy. In the case of central illumination, the significant reduction of the maximum proton energy and the reduced plasma expansion suggest that the laser could not efficiently transfer its energy to electrons, e.g. by means of $\overrightarrow{j}\times \overrightarrow{B}$ - heating^39^ at the steep density gradient. However, when the droplets are irradiated on their sides, the vector of the laser’s electric field has a component pointing into the droplet, and the laser can produce hot electrons more efficiently–most likely via the Brunel mechanism^40^–which afterwards contribute to the proton acceleration process.

**Figure 3:**
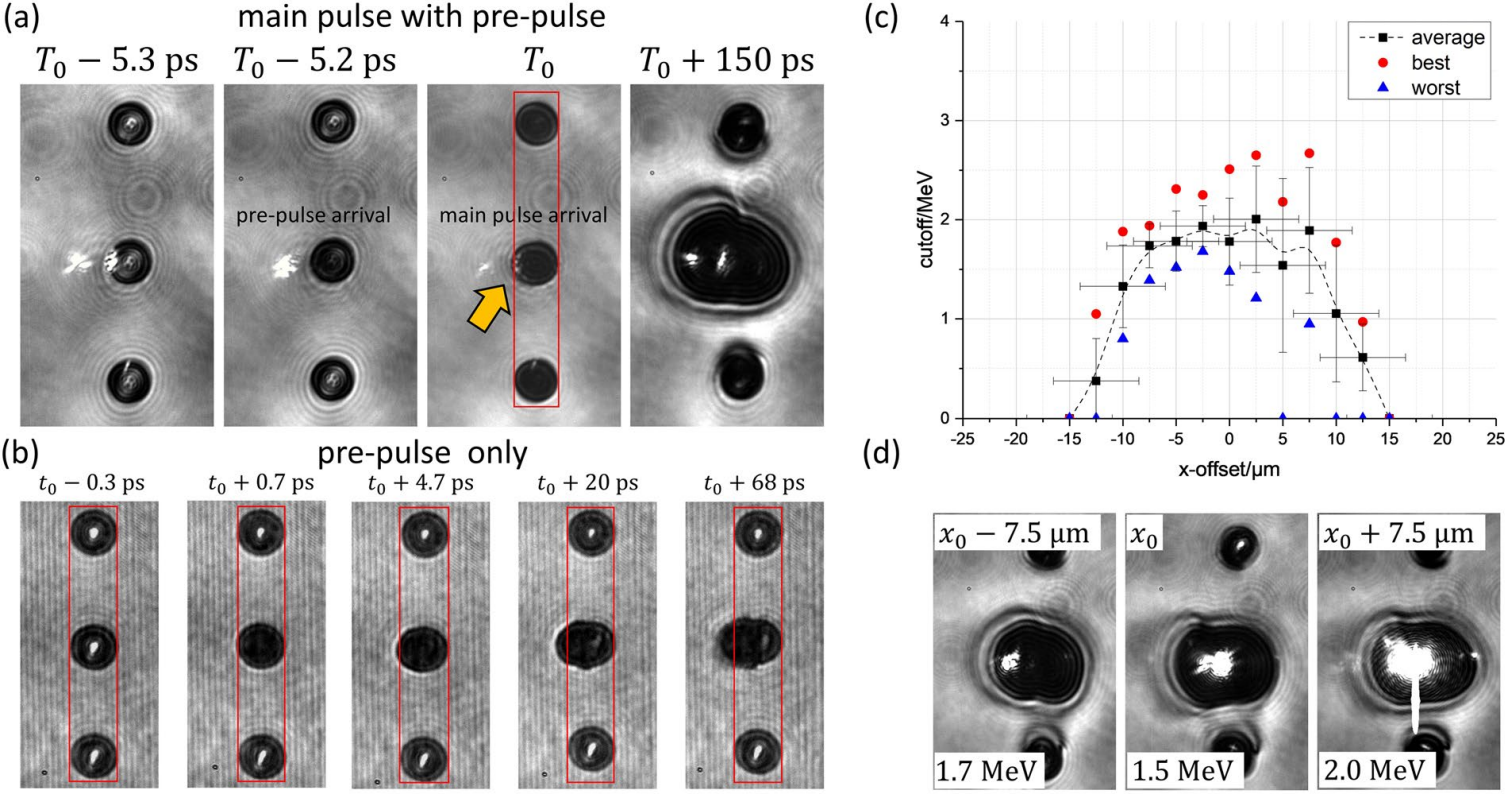
In (**a**) exemplary images of the interaction of the main laser pulse and a pre-pulse with the droplets are shown. The pre-pulse arrives at the time *t*_0_ = *T*_0_ − 5.2 ps, i. e. 5.2 ps before the main pulse. The arrow and the rectangle at *T*_0_ are guides for the eyes to better show that the central droplet has slightly expanded in the direction of the incident laser. Five side-view images for different time steps of the interaction of the pre-pulse alone with the central droplet are shown in (**b**). In (**c**) we show the maximum proton energy in dependence of the droplets’ positions relative to the laser’s focus position for the scan including a pre-pulse. The black squares are averaged values with the corresponding standard deviation of five, consecutive shots at each position, except for *x* = +12.5 μm (6 shots) and *x* = +15 μm (4 shots). Red circles and blue triangles represent the highest and lowest proton energies of the different positions, respectively. The whole scan consists of 65 consecutive shots between *x* = −15 μm and *x* = +15 μm. Again, every single one of these shots is used to calculate the mean values and the standard deviations at their respective positions. Between *x* = −7.5 μm and *x* = +7.5 μm there is only one shot with “0 MeV” (i.e. with no detectable signal on the Thomson parabola). This shot is displayed as the shot with lowest proton energy at *x* = +5 μm (blue triangle) and is the reason for the large error bar, since the other four shots produced energies between 1.8 MeV and 2.2 MeV. This shot triggered a significantly different droplet expansion behavior than the other four shots, which can be seen in Fig. 4 in the Supplemental Material, where these shots are compared to the ones taken at *x* = +12.5 μm. Since the droplets’ absolute positions relative to the laser pulse in the *x*-direction could not be measured directly, it was estimated from the axis of symmetry of the scan. (**d**) shows exemplary images of the illuminated droplet’s expansion for different x-positions 150 ps after the laser droplet interaction took place.

In order to investigate the influence of a pre-plasma in a controlled manner, we introduced a pre-pulse arriving 5.2 ps prior to the main pulse. The timing of the pulse arrivals can be followed in Fig. 3(a). 5.3 ps before the main pulse’s arrival, i.e. before both pre-pulse and main pulse had arrived at the droplet, all droplets were transparent for the probe light. Note that the droplets for this scan, unlike the scan in Fig. 2(a), had a different position on the *x*-axis. Therefore, the light transmitted through the droplets was not imaged as small spots, but as bright, extended, central patches. At the time the pre-pulse arrives (*t*_0_ = *T*_0_ − 5.2 ps), only the central droplet becomes opaque in contrast to the image when the main pulse arrived at *T*_0_ where the central and the two neighbouring droplets are completely opaque due to ionization. In the image showing the arrival of the main pulse, a small pre-plasma expansion on the droplet’s (left) side irradiated by the pre-pulse can be vaguely seen with the help of visual guides (arrow in Fig. 3(a), *T*_0_ image).

With both the main and the pre-pulse applied, the *x*-position of the droplets was again varied, while the maximum proton energy (Fig. 3(c)) and the droplet expansion for *T*_0_ + 150 ps were measured (Fig. 3(d)). The results are quite different when compared to the scan without a pre-pulse (cf. Fig. 2(b,c)). The averaged maximum proton energy stays at a relatively constant level between 1.5 MeV and 2 MeV at positions between *x* = ±7.5 μm, and then decreases as the droplets are moved further away from the laser focus. Thus, the strong dip in Fig. 2(b) has disappeared, probably due to increased electron heating in the pre-plasma. Therefore, the acceleration process is less sensitive to the variation of the droplets’ position along the *x*-axis. which is also reflected in the smaller variation of the proton energies. Simulations show that the energy of electrons increases with increasing scale length^41,42^. A similar behaviour can be observed for the plasma expansion. Here, the shapes of the expanded droplets are quite similar for the different *x*-positions showing an oval-shaped expansion in both laser forward and backward directions without a pronounced bulge shape as in the case without the pre-pulse.

To obtain further information about the pre-plasma the main pulse was blocked using a shutter with a small aperture aligned with the pre-pulse mirror. In Fig. 3(b) exemplary images of the pre-plasma expansion for different time delays are shown. Here, *t*_0_ corresponds to the time of the pre-pulse’s arrival. Before (*t*_0_ − 0.3 ps) and shortly after the pre-pulse arrival (*t*_0_ + 0.7 ps) the central droplet’s size does not change. At later times, the droplet expands mainly in the direction of the incoming laser (i.e. to the left), while no plasma expands from the side facing the Thomson parabola (right side).

These images were used to estimate a 2D-Gaussian pre-plasma with an exponential density profile with the maximum scale length *L*_p,max_ ≈ 0.39 μm ≈ *λ* on the laser axis for the time of arrival of the main laser pulse as input for 2D-PIC simulations (see Supplemental Material for details).

## Simulations and Discussion

2D-PIC simulations were performed using the EPOCH code^43^. The simulation box extended from −15 µm to 15 μm in both axes with 200 cells/μm. The pump laser was modelled as a Gaussian pulse in space and time with a normalized vector potential of *a*_0_ = 2.5, a FWHM pulse duration of *τ* = 42 fs, a FWHM spot size of *d* = 0.6 μm and a central wavelength of *λ* = 0.4 μm. In 2D geometry, the droplets were modelled by disks with a diameter of 5 μm with maximum electron densities of *n*_e,0_ = 30 · *n*_c_. As ions, protons and O^4+^ were included. The number of particles per cell was 20. Due to computational limits, the diameter of the simulated droplets and the laser’s focus had to be reduced with respect to the experiment by a factor of 4 and 2, respectively. To reproduce the experimental results shown in Fig. 2(c) two cases were investigated. First, such a target was centered around (*z*, *x*) = (2.5 μm, 0 μm) resulting in a central incidence of the laser on the droplet representing the case for *x* ≈ 0 µm from Fig. 2(c). For the second case, the target was shifted by its radius to the position (2.5 μm, −2.5 μm) which corresponds to the experimental condition of *x* ≈ ±11.25 μm. To reproduce the proton energy increase for *x* = 0 μm shown in Fig. 3(c) we added a 2D-Gaussian pre-plasma with an exponential density profile with the maximum scale length of *L*_p,max_ = 0.390 μm ≈ *λ* on the laser-axis as estimated. Due to the the unavoidable uncertainties in the experimental determination of the scale length we performed three additional simulations where the maximum scale length was 0.780 μm ≈ 2*λ*, 0.195 μm ≈ 0.5*λ* and 0.100 μm ≈ 0.25*λ* to cover a broad range of scale lengths.

**Figure 4:**
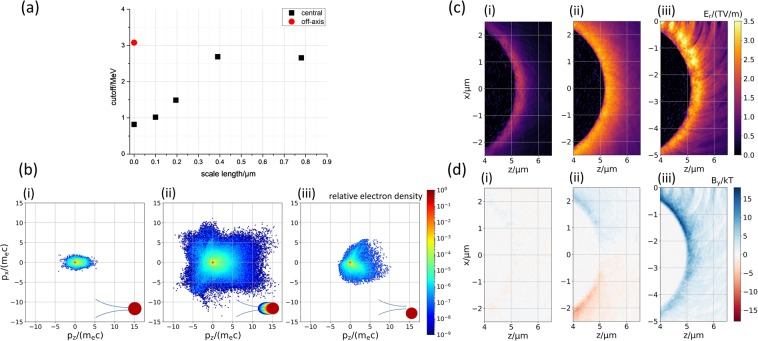
The dependence of the simulated maximum proton energy for different pre-plasma scale lengths and two different irradiation geometries is shown in (**a**). The kinetic energies of protons propagating within an angle of ±1° in the laser forward direction were evaluated from the last recorded time step *T*_0_ + 129 fs. (**b**) Shows the momentum distributions of the electrons normalized to their respective maximum at the time *T*_0_ + 9 fs for (i) the case of the central irradiation of the droplet without pre-plasma, (ii) with pre-plasma with a maximum scale length of *L*_*p*_ = 0.390 μm and (iii) the off-center irradiation without pre-plasma. (**c**) and (**d**) show the radial electric field *E*_*r*_ and the magnetic field component *B*_*y*_ at the droplet’s right side for a time *T*_0_ + 29 fs. *T*_0_ denotes the time at which the main pulse peak arrives at its focus at (*z* = 0, *x* = 0). As a consequence, the droplet’s top side was irradiated in this 2D-geometry for the case of grazing incidence. A sketch of the irradiation conditions is shown in (**a**) in the lower right corner. The  entire droplet with the magnetic field in y-direction, including the laser field, is also shown for *T*_0_ + 29 fs, *T*_0_ + 49 fs and *T*_0_ + 69 fs in the Supplemental Material as Fig. 7.

In Fig. 4(a) the maximum proton energy of forward accelerated protons at the end of the simulation run for the two different irradiation geometries and the four different pre-plasma profiles is shown. It can be seen that the off-axis irradiation produces a nearly four times higher proton energy than the central irradiation of the droplet, as long as no pre-plasma is present. The simulations with the various scale lengths under normal incidence show that the presence of a pre-plasma always produces a rise in the proton energies compared to the case without pre-plasma. The proton energy increases with increasing scale length up to *L*_p,max_ = 0.390 μm and does not increase further for *L*_p,max_ = 0.780 μm. However, for off-axis irradiation without a pre-plasma present the maximum proton energy is still slightly higher than for all scale lengths and normal laser incidence. Therefore, the simulation reproduces well the experimental findings. One should note, however, that for the off-axis illumination case even higher proton energies are emitted under different angles in the simulation (see the protons’ distribution in the phase space in Fig. 2(f) in the Supplemental Material).

To study the reasons for the different maximum proton energies we investigated the electrons’ phase space (Fig. 4(b)) and the electric field behind the droplet (Fig. 4(c)) for (i) the normal incidence case without a pre-plasma, (ii) normal incidence with a pre-plasma with the maximum scale length of *L*_p,max_ ≈ *λ* and (iii) the case of off-axis irradiation without a pre-plasma.

When comparing (b_i_) and (b_ii_), it is obvious that electrons are heated much more when a pre-plasma is present. The electrons in (b_i_) are only slightly accelerated in the forward direction, since the field of the laser is strongly attenuated at the outer edge of the droplet and can hardly heat electrons due to the high electron density inside the droplet which results in a small skin depth^44^. For the simulation with a scale length of ≈ *λ* shown in (b_ii_) the density distribution of electrons in the momentum space shows that a large number of electrons get strongly accelerated in all directions from the pre-plasma resulting in a larger number of hot electrons at the droplet’s back and therefore a stronger electric field driving the proton acceleration. However, the electric field is still somewhat weaker than in the case of off-axis irradiation (compare (c_ii_) with (c_iii_)) although in this case less hot electrons are produced (b_iii_).

This stronger electric field in the case of off-axis irradiation can be explained as follows^31,32^. Here, the laser’s electric field directly points into the droplet and can therefore strongly heat electrons near the surface. Many of these electrons get additionally accelerated in forward direction and travel just outside the droplet close to its surface. This current of hot electrons induces a return current of cold electrons just below the surface of the droplet. These two opposing currents generate a strong magnetic field in the positive *y*-direction localized at the surface (Fig. 4 (d_iii_)), where the resulting Lorentz force pushes the hot electrons away from the droplet. The charge separation field which is localized a little further outwards accelerates the electrons back towards the droplet. Together the two fields produce a localized population of hot electrons travelling along the surface. Such surface electrons have already been observed or simulated when using planar targets^33–35^. Also for the case of droplets, this very localized electron sheath is responsible for the stronger electric field accelerating protons to higher kinetic energies than in the other cases. An example for such a confined hot electron is shown in the Supplementary Material (Fig. 6).

## Summary

In summary, we have investigated the interaction of ultra-high contrast laser pulses at 400 nm with water microdroplets, with and without an additional pre-pulse, by means of an optical probe and the measurement of maximum proton energies as well as 2D-PIC simulations.

The optical probe not only allowed us to observe the expansion of the droplets on a picosecond timescale, providing additional insight into how a droplet was irradiated by the main laser pulse, but also allowed us to estimate the plasma expansion induced by an additional pre-pulse. The pre-plasma changed the interaction in a way that the main laser pulse’s conversion into hot electrons was enhanced which eventually led to higher proton energies in the forward direction when the droplets were irradiated under normal incidence.

However, when the droplets were shifted about their radius along the laser’s polarization direction–without a pre-plasma present–even slightly higher proton energies in the experiment, for single shots, as well as in the corresponding simulations were produced. Here, the off-axis irradiation of a droplet causes a hot electron current to flow along the surface of the droplet, generating electric and magnetic fields that in turn confine the current close to the surface. This confinement of the current in combination with the limited volume of the targets enhances the electric field responsible for proton acceleration. This behavior is only possible for targets with spherical geometry such as droplets because the surface current cannot escape the interaction region in this case.

However, by generating a pre-plasma, the acceleration process becomes more stable, because the sensitivity on the actual droplet position is reduced. Since most applications require stable proton sources, laser pulses with carefully adjusted TIC may be a good choice. Therefore, the investigation of laser pulses with different pre-pulses, or different TIC, is useful to achieve high proton energies with high reproducibility.

Thus, we have shown that the combination of water microdroplets irradiated at 2*ω* frequencies and probing with off-harmonic wavelengths has great potential for the investigation of laser-plasma interactions. Furthermore, the ease of droplet production, their synchronizability with the pump laser and availability at high repetition rates demonstrate that droplets are a promising target for laser-driven proton acceleration as well as for secondary radiation sources^45^.

## Supplementary information

Supplementary figures

## Data Availability

The datasets generated and analyzed during the current study are available from the corresponding authors on reasonable request. The simulations were analyzed with open-source software^46–49^.

## References

[1] Macchi A, Borghesi M, Passoni M (2013). Ion acceleration by superintense laser-plasma interaction. Rev. Mod. Phys..

[2] Daido H, Nishiuchi M, Pirozhkov AS (2012). Review of laser-driven ion sources and their applications. Reports on Prog. Phys..

[3] Clark E (2000). Energetic heavy-ion and proton generation from ultraintense laser-plasma interactions with solids. Phys. Rev. Lett..

[4] Snavely RA (2000). Intense high-energy proton beams from petawatt-laser irradiation of solids. Phys. Rev. Lett..

[5] Wilks S (2001). Energetic proton generation in ultra-intense laser–solid interactions. Phys. Plasmas.

[6] Barberio M (2018). Laser-accelerated particle beams for stress testing of materials. Nat. Commun..

[7] Barberio M, Veltri S, Scisciò M, Antici P (2017). Laser-accelerated proton beams as diagnostics for cultural heritage. Sci. Reports.

[8] Busold S (2014). Shaping laser accelerated ions for future applications–the light collaboration. Nucl. Instruments Methods Phys. Res. Sect. A: Accel. Spectrometers, Detect. Assoc. Equip..

[9] Jahn D (2018). First application studies at the laser-driven light beamline: Improving proton beam homogeneity and imaging of a solid target. Nucl. Instruments Methods Phys. Res. Sect. A: Accel. Spectrometers, Detect. Assoc. Equip..

[10] Mackinnon AJ (2004). Proton radiography as an electromagnetic field and density perturbation diagnostic (invited). Rev. Sci. Instruments.

[11] Hanton F (2019). Dna dsb repair dynamics following irradiation with laser-driven protons at ultra-high dose rates. Sci. reports.

[12] Ledingham K, Bolton P, Shikazono N, Ma C-M (2014). Towards laser driven hadron cancer radiotherapy: A review of progress. Appl. Sci..

[13] Gao, Y. *et al*. An automated, 0.5 Hz nano-foil target positioning system for intense laser plasma experiments. *High Power Laser Sci. Eng*. **5**, E12 (2017).

[14] McKenna P (2007). Lateral electron transport in high-intensity laser-irradiated foils diagnosed by ion emission. Phys. Rev. Lett..

[15] Paasch-Colberg T (2011). New method for laser driven ion acceleration with isolated, mass-limited targets. Nucl. Instruments Methods Phys. Res. Sect. A: Accel. Spectrometers, Detect. Assoc. Equip..

[16] Sokollik T (2010). Laser-driven ion acceleration using isolated mass-limited spheres. New J. Phys..

[17] Ostermayr TM (2016). Proton acceleration by irradiation of isolated spheres with an intense laser pulse. Phys. Rev. E.

[18] Hilz P (2018). Isolated proton bunch acceleration by a petawatt laser pulse. Nat. Commun..

[19] Busch S (2003). Ion acceleration with ultrafast lasers. Appl. Phys. Lett..

[20] Schnürer M (2005). Ion acceleration with ultrafast laser driven water droplets. Laser Part. Beams.

[21] Sokollik T (2009). Directional laser-driven ion acceleration from microspheres. Phys. Rev. Lett..

[22] Ter-Avetisyan S (2004). Spectral dips in ion emission emerging from ultrashort laser-driven plasmas. Phys. Rev. Lett..

[23] Ter-Avetisyan S (2006). Quasimonoenergetic deuteron bursts produced by ultraintense laser pulses. Phys. Rev. Lett..

[24] Obst L (2017). Efficient laser-driven proton acceleration from cylindrical and planar cryogenic hydrogen jets. Sci. Reports.

[25] Ziegler T (2018). Optical probing of high intensity laser interaction with micron-sized cryogenic hydrogen jets. Plasma Phys. Control. Fusion.

[26] Sävert A (2015). Direct observation of the injection dynamics of a laser wakefield accelerator using few-femtosecond shadowgraphy. Phys. Rev. Lett..

[27] Schwab M (2013). Few-cycle optical probe-pulse for investigation of relativistic laser-plasma interactions. Appl. Phys. Lett..

[28] Downer M, Zgadzaj R, Debus A, Schramm U, Kaluza M (2018). Diagnostics for plasma-based electron accelerators. Rev. Mod. Phys..

[29] Jäckel O (2010). All-optical measurement of the hot electron sheath driving laser ion acceleration from thin foils. New J. Phys..

[30] Adolph D (2017). Real-time, single-shot, carrier-envelope-phase measurement of a multi-terawatt laser. Appl. Phys. Lett..

[31] Nakamura T, Kato S, Nagatomo H, Mima K (2004). Surface-magnetic-field and fast-electron current-layer formation by ultraintense laser irradiation. Phys. Rev. Lett..

[32] Nakamura T, Mima K, Sakagami H, Johzaki T (2007). Electron surface acceleration on a solid capillary target inner wall irradiated with ultraintense laser pulses. Phys. Plasmas.

[33] Li Y (2006). Observation of a fast electron beam emitted along the surface of a target irradiated by intense femtosecond laser pulses. Phys. Rev. Lett..

[34] Psikal J, Tikhonchuk V, Limpouch J, Klimo O (2010). Lateral hot electron transport and ion acceleration in femtosecond laser pulse interaction with thin foils. Phys. Plasmas.

[35] Schumaker W (2013). Ultrafast electron radiography of magnetic fields in high-intensity laser-solid interactions. Phys. review letters.

[36] Bierbach J (2012). Generation of 10 mw relativistic surface high-harmonic radiation at a repetition rate of 10 hz. New J. Phys..

[37] Yeung M (2017). Experimental observation of attosecond control over relativistic electron bunches with two-colour fields. Nat. Photonics.

[38] Kahaly S (2013). Direct observation of density-gradient effects in harmonic generation from plasma mirrors. Phys. Rev. Lett..

[39] Kruer W, Estabrook K (1985). J × b heating by very intense laser light. The Phys. Fluids.

[40] Brunel F (1987). Not-so-resonant, resonant absorption. Phys. Rev. Lett..

[41] Paradkar B (2011). Numerical modeling of fast electron generation in the presence of preformed plasma in laser-matter interaction at relativistic intensities. Phys. Rev. E.

[42] Wu D, Krasheninnikov S, Luan S, Yu W (2016). Identifying the source of super-high energetic electrons in the presence of pre-plasma in laser–matter interaction at relativistic intensities. Nucl. Fusion.

[43] Arber T (2015). Contemporary particle-in-cell approach to laser-plasma modelling. Plasma Phys. Control. Fusion.

[44] Wilks SC, Kruer WL (1997). Absorption of ultrashort, ultra-intense laser light by solids and overdense plasmas. IEEE J. Quantum Electron..

[45] Palmer C (2018). Paving the way for a revolution in high repetition rate laser-driven ion acceleration. New J. Phys..

[46] Walt Svd, Colbert SC, Varoquaux G (2011). The numpy array: a structure for efficient numerical computation. Comput. Sci. & Eng..

[47] Hunter JD (2007). Matplotlib: A 2d graphics environment. Comput. In Sci. & Eng..

[48] Pérez, F. & Granger, B. E. Ipython: a system for interactive scientific computing. *Comput. Sci. & Eng*. **9** (2007).

[49] Kuschel, S. *et al*. *The open-source particle in cell postprocessor*. http://github.com/skuschel/postpic (2018).

