# Supplementary material for: Characterization of laser-driven proton acceleration from water microdroplets
Source: Sci Rep. 2019 Nov 20;9:17169. doi: 10.1038/s41598-019-53587-3 (PMC6868211; doi:10.1038/s41598-019-53587-3)
Supplement: Supplementary file 1 — Supplementary figures [file 41598_2019_53587_MOESM1_ESM.pdf]

Supplemental Material for: Characterization of laser-driven proton acceleration from water microdroplets

Georg A. Becker[1,*], Matthew B. Schwab[1], Robert Lötzsch[1], Stefan Tietze[1,2], Diethard Klöpfel[2], Martin Rehwald[3,4], Hans-Peter Schlenvoigt[3], Alexander Sävert[1,2], Ulrich Schramm[3,4], Matt Zepf[1,2], and Malte C. Kaluza[1,2]

[1]Institut für Optik und Quantenelektronik, Friedrich-Schiller-Universität Jena, Max-Wien-Platz 1, D-07743 Jena, Germany
[2]Helmholtz-Institut Jena, Fröbelstieg 3, D-07743 Jena, Germany
[3]Helmholtz-Zentrum Dresden-Rossendorf (HZDR), Bautzner Landstraße 400, D-01328 Dresden, Germany
[4]Technische Universität Dresden, D-01062 Dresden, Germany
[*]Correspondence and requests for materials should be adressed to G.A.B. (email: georg.becker@uni-jena.de) or M.C.K. (email: malte.kaluza@uni-jena.de)

Estimation of the pre-plasma profile

The side-view images from the measurements with pre-pulse only (see Fig. 3 (b) in the main article for some exemplary images) were used to estimate a pre-plasma profile for the time of the arrival of the main pulse in the scan where both main pulse and pre-pulse were applied in order to obtain a two-dimensional pre-plasma profile for the two-dimensional particle-in-cell (2D-PIC) simulations. This was done with the following estimates. It was assumed that the maximum number of electrons released by the pre-pulse was eight per water molecule (six from oxygen and two from hydrogen), resulting in a maximum electron density of $n_{\mathrm{e},0} \approx 123 \cdot n_\mathrm{c}$ at the initial droplet surface, where n_c is the critical density for the probe wavelength of $\lambda = 0.71\,\mu\mathrm{m}$. The expansion front was assumed to have a density of $\sim 1 \cdot n_\mathrm{c}$. The evolution of the expansion was measured for several shots and timesteps up to $\sim 20\,\mathrm{ps}$ after the pre-pulse's arrival (Fig. 1). Additionally the expansion was measured under different angles between 0° and 90° with respect to the incoming laser direction. The pre-plasma was assumed to have an exponential density profile, on all measured axes, between $n_{\mathrm{e},0}$ and n_c with $n_e(x) = n_{\mathrm{e},0} \cdot \exp(-x/L)$ where L denotes the pre-plasma scale length. From these measurements, a pre-plasma profile could be estimated in which the pre-plasma expands in directions between 0° and $\sim \pm 60^\circ$ with the maximum scale length $L_{\mathrm{p,max}} = 0.39\,\mu\mathrm{m}$ on the laser axis. Due to the unavoidable uncertainties in these measurements, we performed additional 2D-PIC simulations with pre-plasma profiles with twice, half and a quarter of the experimentally deduced maximum scale length.

Determination of the effective magnetic field in the Thomson parabola

The effective magnetic field was determined in a different experimental campaign by placing CR-39 nuclear track detectors shielded with aluminum foils of different thicknesses inside the Thomson parabola spectrometer. In this experiment, higher proton energies (between 2 MeV and 10 MeV) were achieved. The distances y between the zero deflection point and the low energy cutoffs E_kin of the proton traces, which are the proton energies blocked by the used aluminum foils, are determined by the magnetic field B. With the known distance d between the CR-39 detector and the magnet (which was 150 mm during that calibration experiment), as well as the length L of the magnet, it is possible to obtain the magnetic field strength from a fit with the function $y = R - \sqrt{R^2 - L^2} + \frac{Ld}{\sqrt{R^2 - L^2}}$, where $R = \frac{m_p v}{eB} = \frac{\sqrt{2E_\mathrm{kin} m_p}}{eB}$ is the Larmor radius. For $R \gg L$ this can be simplified to $y = \frac{eBL}{m_p v}\left(\frac{L}{2} + d\right)$. This formula can be found, for example, in the article by Choi et al.[1]. With the values for B, L, d given in the Methods section in the main article, one can obtain the parabola's dispersion curve and with the given aperture the energy resolution.

Error bars and stability

The error bars for the position along the x-axis (Fig. 2 (b) and Fig. 3 (c) in the main article) include the estimated maximum positioning error of the motor used to translate the nozzle, which was $\sim \pm 2.5\,\mu m$. The main laser's pointing at full intensity could not be determined. However, the beam pointing stability of the laser pulse under reduced-energy conditions was measured in the far-field with a standard deviation of $\sim \pm 1.4\,\mu m$. An additional contribution to the entire error is given by the stability of the droplets. Since the droplets' position along the x-axis could not be determined exactly, the stability along the z-axis (i.e. in laser-forward direction) was measured, which should be comparable to the stability along the x-axis. For all 230 shots included in Fig. 2 (b) and Fig. 3 (c) of the main article the standard deviation is $\sim \pm 0.5\,\mu m$. Therefore, the combination of all three errors along the x-axis (one systematic and two statistical) is $\sim \pm 4\,\mu m$.

The error bars for the maximum proton energies in Fig. 2 (b) and Fig. 3 (c) of the main article are the standard deviations of all shots taken at each position. The Thomson parabola's resolution is not included in the error bars, but is $\Delta E/E \approx 10\,\%$ for an energy of 2 MeV, as stated in the "Methods" section of the main article. The shots with no signal above the low-energy cutoff of the Thomson parabola (0.4 MeV) were included with an energy of 0 MeV in the calculation of the mean values and standard deviations. For example, in Fig. 3 (c) in the main article between $x = -7.5\,\mu m$ and $x = +7.5\,\mu m$, there is only one shot with 0 MeV (i.e., with no detectable signal on the Thomson parabola). This shot is displayed in the plot as the shot with the lowest proton energy at $x = +5\,\mu m$ (blue triangle) and is the reason for the large error bar since the other four shots produced energies between 1.8 MeV and 2.2 MeV. The side-view images of all five shots taken at $x = +5\,\mu m$ are shown in Fig. 4 (a) with the corresponding shot number (the scan consists of 65 shots) and the maximum proton energy. The irradiated droplet for the shot with 0 MeV (#44) has expanded much less compared to the other four shots taken at this position. For comparison, Fig. 4 (b) shows five of the six shots taken at $x = +12.5\,\mu m$, where the droplet was intentionally misaligned. Here, all five shots had maximum proton energies at or below 0.8 MeV, where the plasma expansion is significantly reduced compared to the four "good" shots in Fig. 4 (a). A closer look reveals that the expanded droplet in shot #44 ($x = +5\,\mu m$) is a bit smaller than the droplet in shot #59 ($x = +12.5\,\mu m$, 0.5 MeV, slightly above the low-energy cutoff) and a bit larger than the droplet in shot #60 (no signal above the low-energy cutoff) and shows a comparable shape. It might be that for shot #44 the laser did hit the droplet in a comparable way as in shots #59 or #60, which means the laser did only "partially" hit the droplet. However, since no online diagnostic for the full intensity focus was implemented in the experiment, it was not possible to verify this assumption.

In Fig. 5 of this supplemental material, MCP spectra for the case without (a) and with the applied pre-pulse (b) are shown. In (a), the two graphics (left) for central irradiation of the droplets ($x = -1.25\,\mu m$) include 8 spectra (two shots were below the low-energy cutoff), while the graphics (right) for grazing incidence ($x = +11.25\,\mu m$) include the spectra for all 10 shots taken at this position. It is obvious that for most shots the maximum proton energies as well as the proton numbers are larger for $x = +11.25\,\mu m$. For the pre-plasma case (b), all spectra obtained between $x = -5\,\mu m$ and $x = 2.5\,\mu m$ are shown (5 shots for each position). Here, the maximum proton energies and particle numbers are more stable than compared to (a).

References

1. Choi, I. *et al.* Ion spectrometer composed of time-of-flight and thomson parabola spectrometers for simultaneous characterization of laser-driven ions. *Rev. Sci. Instruments* **80**, 053302 (2009).

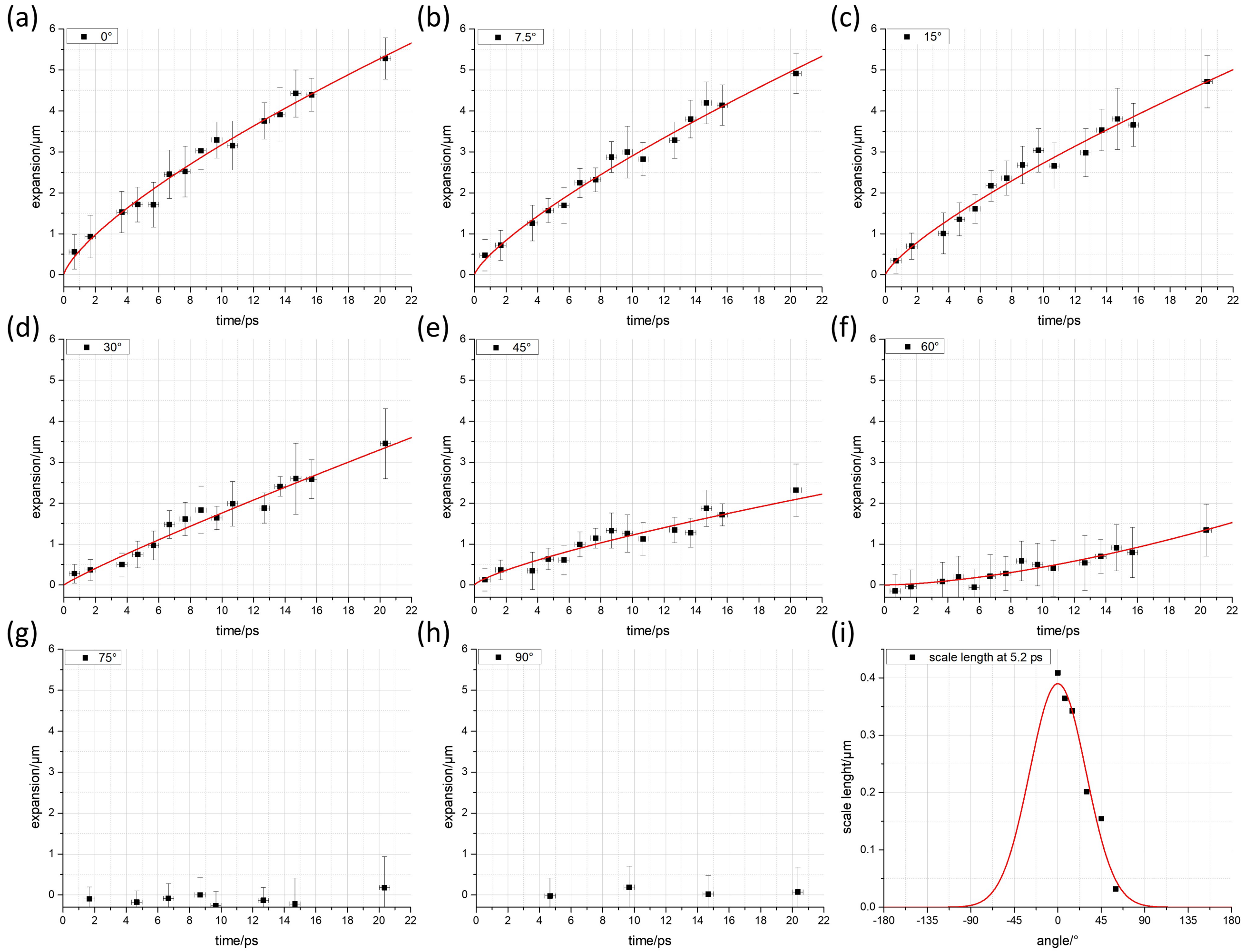

Figure 1. I (a) - (h), the droplet expansion is shown as a function of the time after the pre-pulse arrival for different angles θ with respect to the axis of the incoming laser. The red lines are power function fits from which the pre-plasma expansion at the main pulse arrival was extrapolated (5.2 ps). From these measurements, the pre-plasma scale length could be determined as a Gaussian function of θ for the time of the main pulse arrival as shown in (i).

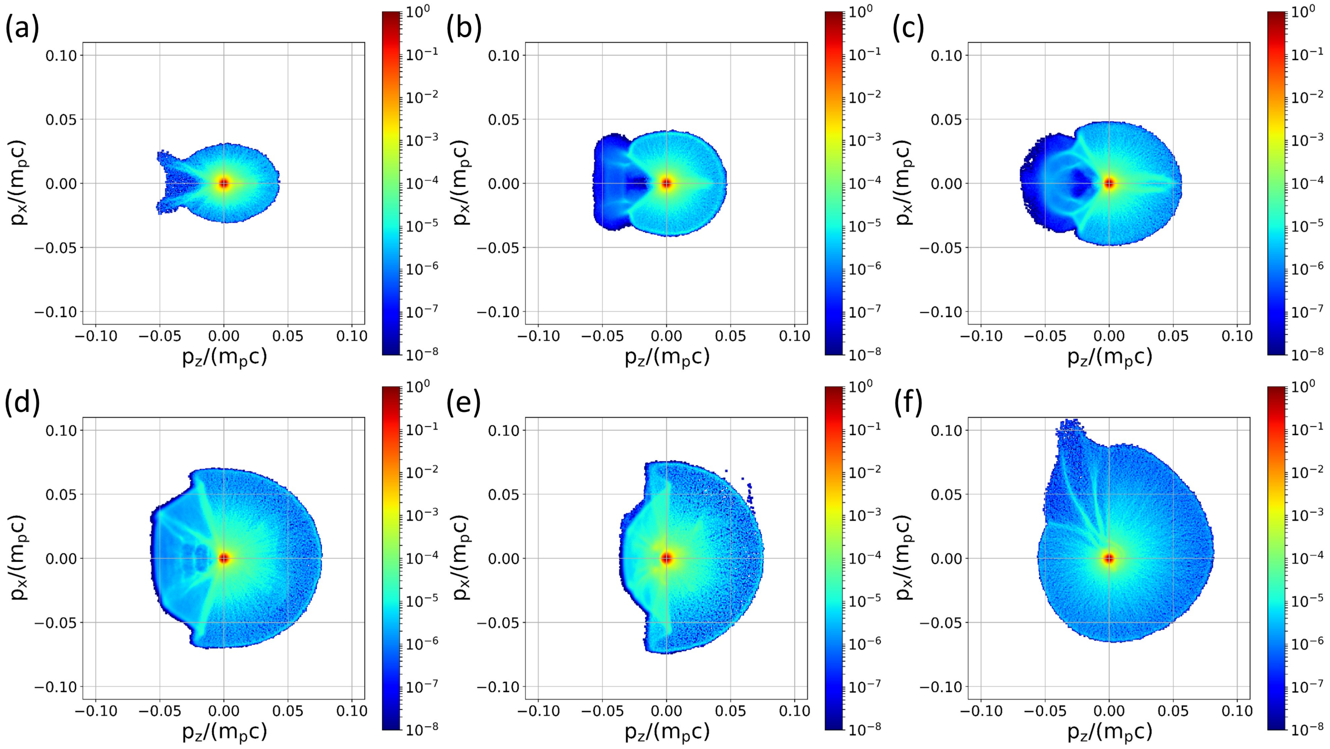

Figure 2. The images show the momentum distributions for the protons normalized to their respective maximum at the end of the simulation at $T_0 + 129\,\mathrm{fs}$. (a) - (e) show the protons' momentum distribution for normal laser incidence (a) without a pre-plasma present, with a Gaussian pre-plasma with a maximum scale length of (b) $L_{\mathrm{p,max}} = 0.100\,\mu\mathrm{m}$, (c) $L_{\mathrm{p,max}} = 0.195\,\mu\mathrm{m}$, (d) $L_{\mathrm{p,max}} = 0.390\,\mu\mathrm{m}$, (e) $L_{\mathrm{p,max}} = 0.780\,\mu\mathrm{m}$. (f) shows the protons' momentum distribution for the off-axis irradition of a droplet without a pre-plasma.

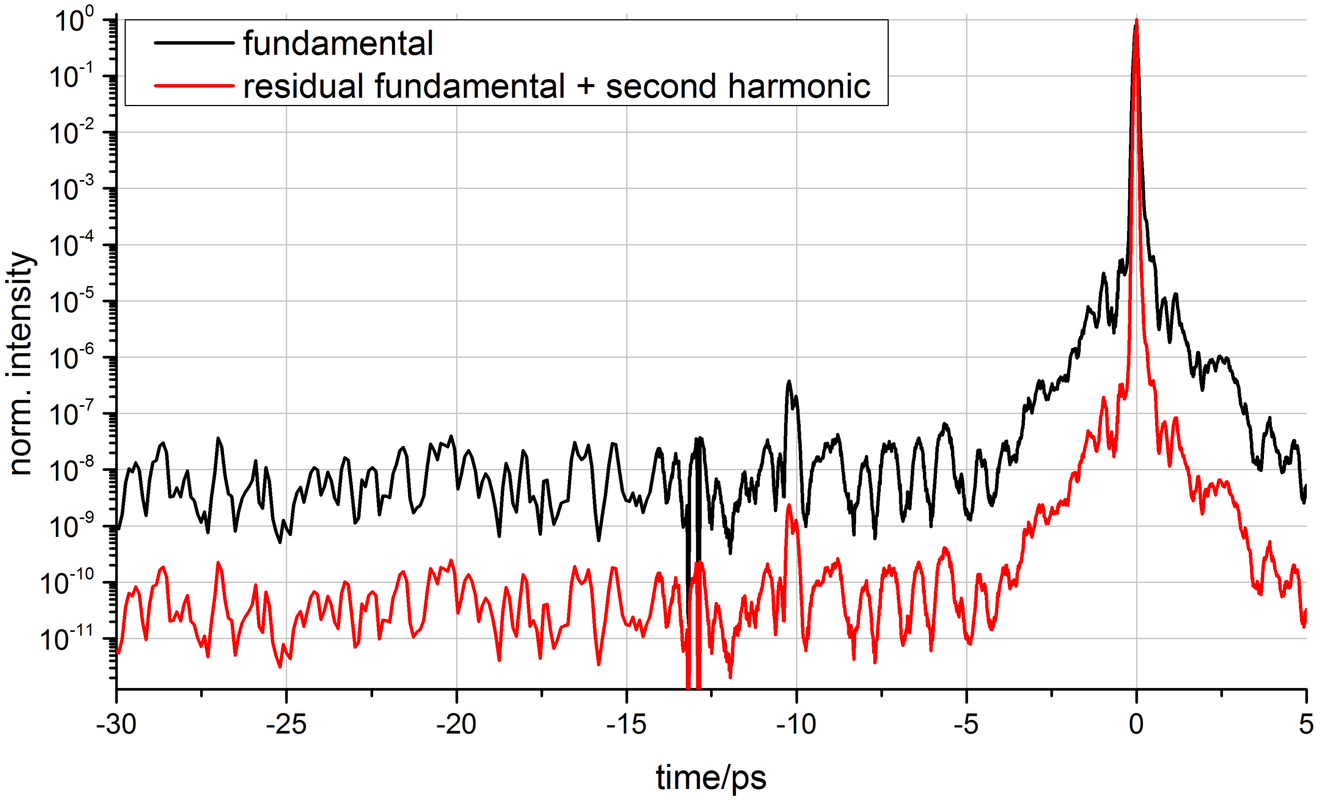

Figure 3. The black line shows the temporal intensity contrast (TIC) of the JETI 40 laser system for the fundamental wavelength. The red line is the calculated TIC consisting of the second harmonic and the remaining fundamental radiation. It was calculated with a conversion efficiency of $\sim 16\,\%$, an extinction ratio of ~ 250 for the two dichroic mirrors and with the assumption that the focus of the fundamental was about a factor of 4 larger than the focus of the second harmonic.

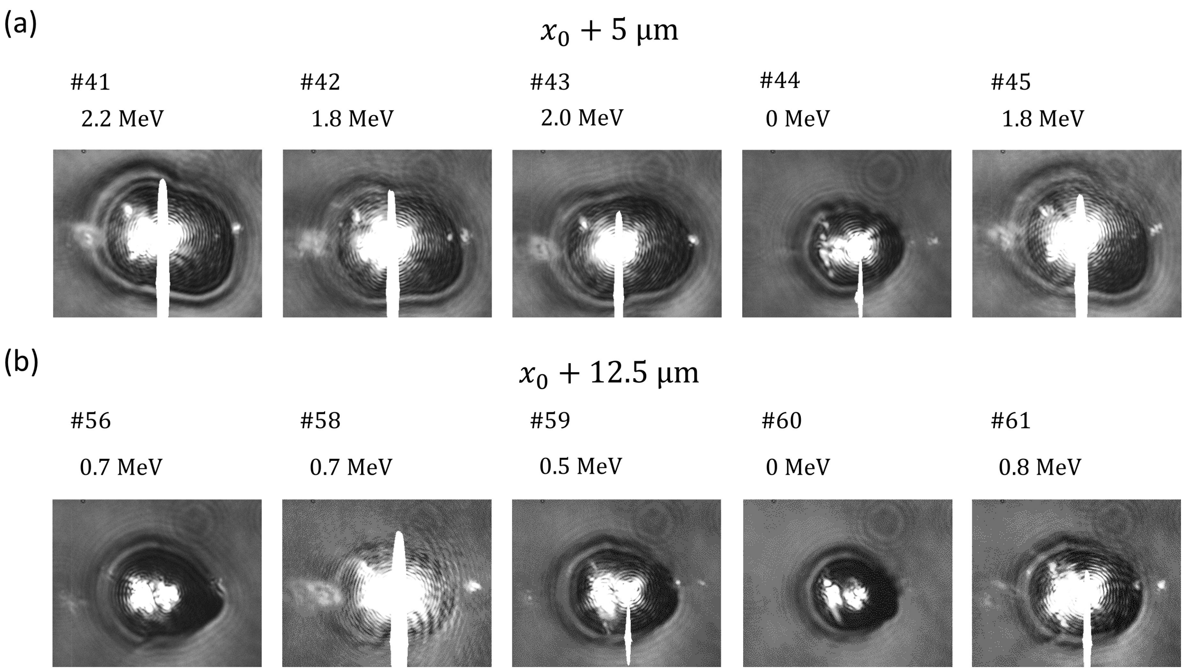

Figure 4. (a) shows all five side-view images recorded at the position $x = +5\,\mu\text{m}$ for the scan shown in Fig. 3 (c) in the main article. The expansion is captured $T_0 + 150\,\text{ps}$ after the laser droplet interaction. The number above the images is the shot number, within a scan consisting of a total 65 consecutive shots. (b) shows 5 of the 6 side-view images captured at $x = +12.5\,\mu\text{m}$. Shot #57 is not shown, since the camera overload was stronger than for shot #58.

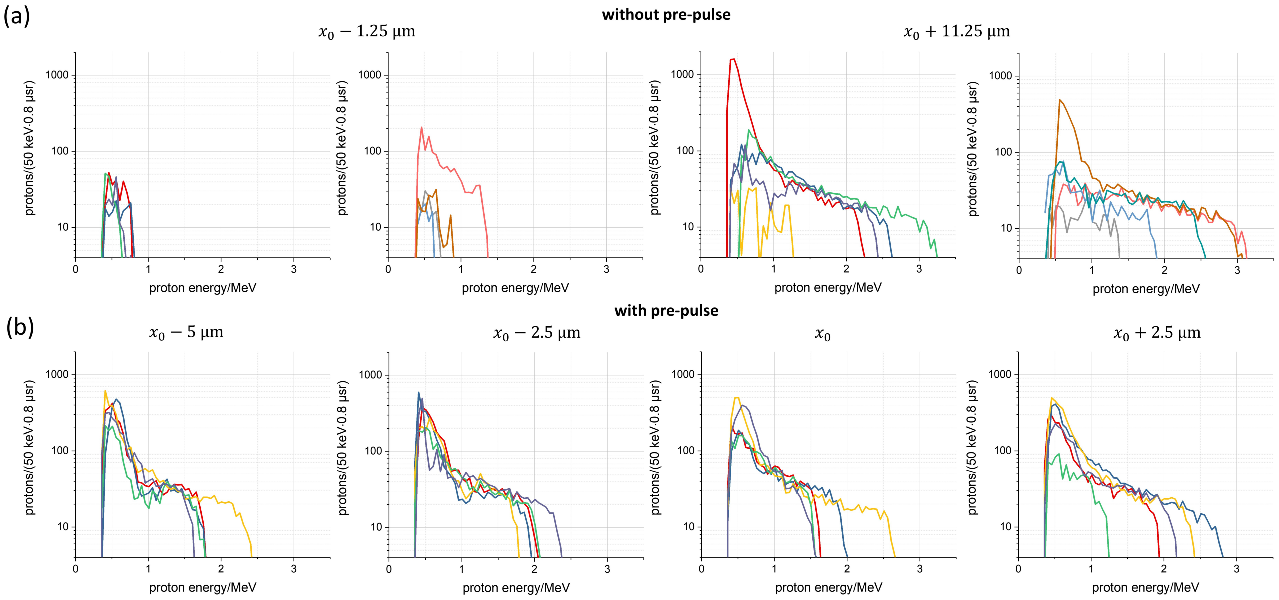

Figure 5. The figure shows MCP spectra for two x-positions per contrast setting (see Fig. 2 (b) and Fig. 3 (c) in the main article). The left images of (a) show 8 spectra of the 10 shots taken at $x = -1.25\,\mu\text{m}$, where the other two shots did not produce any MCP signal above the low-energy cutoff of the Thomson parabola. However, these shots were included as 0 MeV in the calculation of the mean value and standard deviation for Fig. 2 (b) in the main article. The right images show the spectra of all 10 shots taken at $x = +11.25\,\mu\text{m}$. In (b) all 20 spectra out of 20 consecutive shots, obtained between $x = -5\,\mu\text{m}$ and $x = 2.5\,\mu\text{m}$ (5 shots each position), for the scan with the applied pre-pulse are shown (Fig. 3 (c) in the main article).

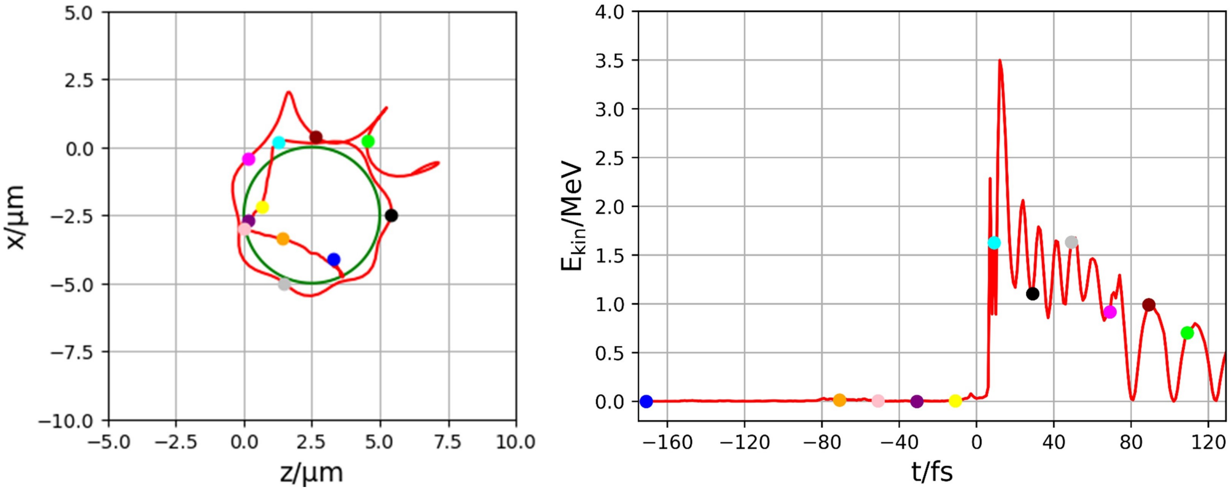

Figure 6. An example of the trajectory of a single, exemplary hot electron (left) along with the evolution of its kinetic energy (right) is shown. The blue dot denotes the start of the simulation, which is 171 fs before the main pulse peak arrives at $(z = 0\,\mu\text{m}, x = 0\,\mu\text{m})$, therefore $t = T_0 - 171$ fs. The orange dot is at $t = T_0 - 71$ fs and afterwards each dot marks the position and the kinetic energy after an additional 20 fs. Up to the time around $t = T_0 + 9$ fs (cyan dot, which is the same time as for the plot of the electrons' momentum distribution shown in Fig. 4 in the main article), the electron travels inside the droplet to the point, where the laser has extracted electrons and caused a charge difference inside the droplet. Then the laser pulls the electron out of the droplet and accelerates it in positive z-direction. From then on, the electron performs oscillations close to the droplet's surface caused by both the electric and the magnetic field. The black dot is at $t = T_0 + 29$ fs, the time for which the electric and magnetic fields are shown in Fig. 4 in the main article. Towards the end of the simulation, the amplitude of the oscillation grows larger, since the electric field is localized further away from the initial droplet surface due to plasma expansion, especially at the laser incidence region, where the plasma expansion is strongest.

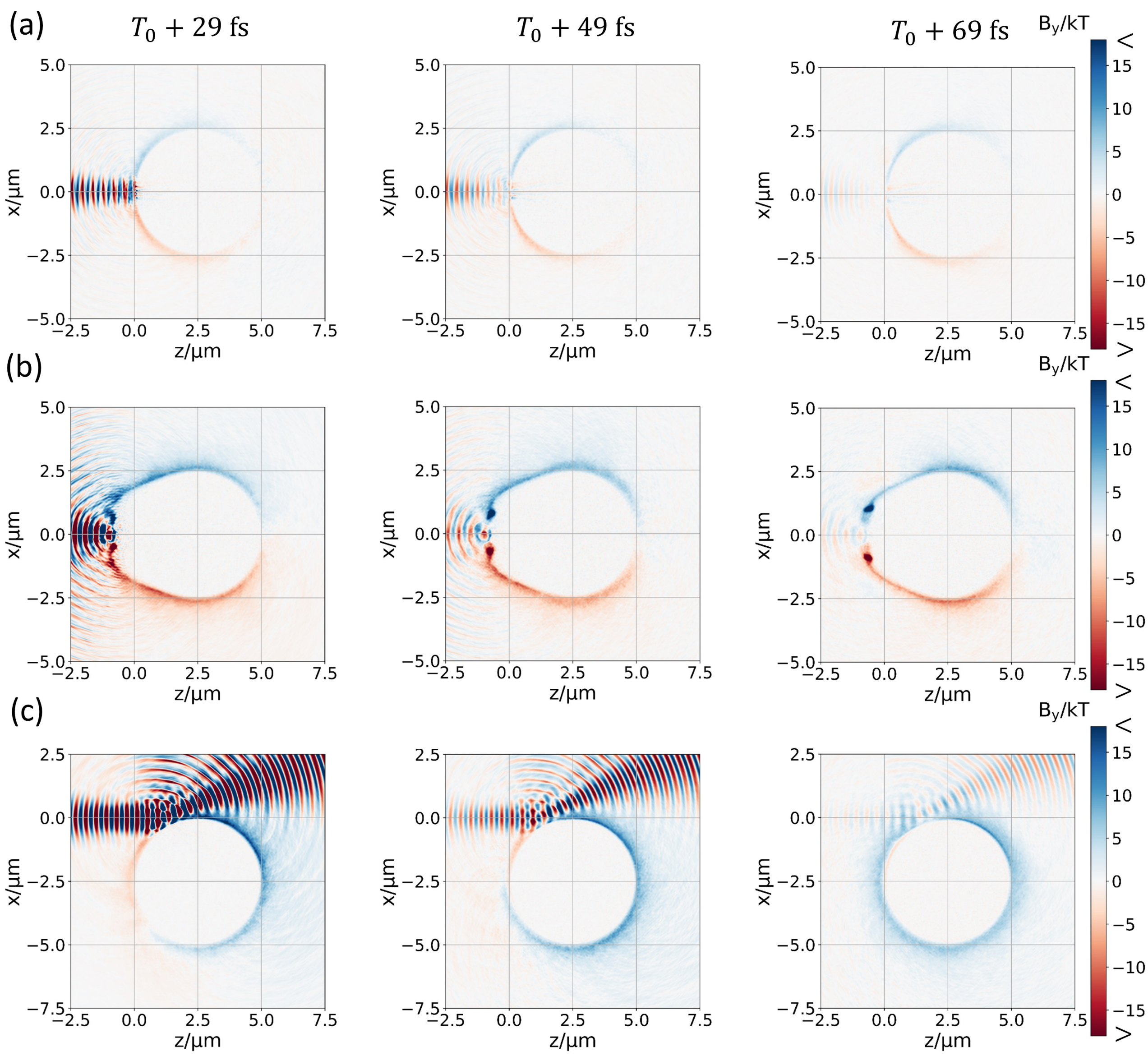

Figure 7. This figure shows the magnetic field around the entire droplet, including the laser's field, for all three irradiation cases considered in Fig. 4 in the main article for three different time steps. (a) shows the magnetic field for normal laser incidence without a pre-plasma, (b) for normal laser incidence with a Gaussian-shaped pre-plasma with a maximum scale length of $L_{\mathrm{p,max}} = 0.390\,\mu\mathrm{m}$ on axis and (c) for off-axis irradiation without a pre-plasma.

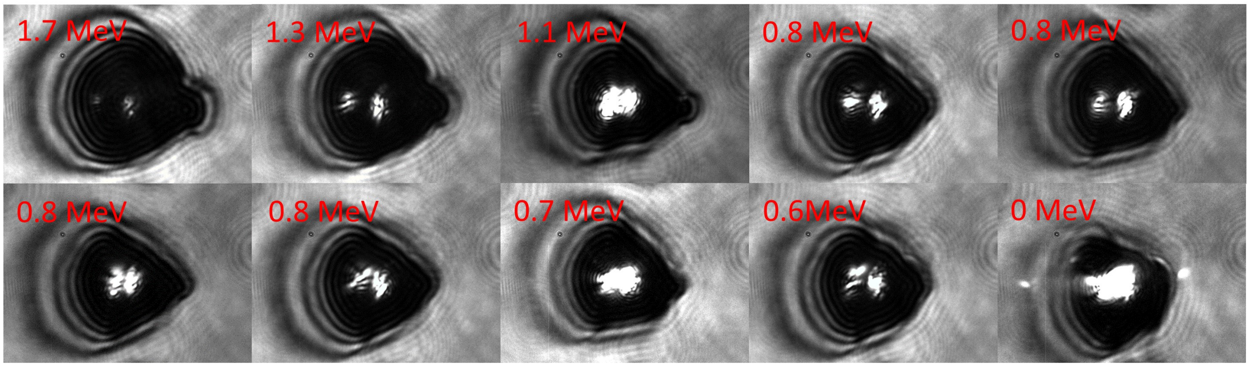

Figure 8. The figure shows all side-view images for the shots taken at $x = +1.25\,\mu m$ without pre-pulse (Fig. 2 (b) in the main article). The expansion is captured at $t = T_0 + 150\,ps$. The images were sorted according to the maximum proton energy achieved in the respective shot.
